# Characteristics of the pulmonary microbiota in patients with mild and severe pulmonary infection

**DOI:** 10.3389/fcimb.2023.1227581

**Published:** 2023-10-12

**Authors:** Danting Zhan, Dan Li, Ke Yuan, Yihua Sun, Lijuan He, Jiacheng Zhong, Lingwei Wang

**Affiliations:** ^1^ Shenzhen Institute of Respiratory Diseases, Shenzhen People’s Hospital, Guangdong, China; ^2^ BGI Genomics, Shenzhen, China

**Keywords:** pulmonary infection, pulmonary microbiota, severity, network, biomarker

## Abstract

**Background:**

Lung infection is a global health problem associated with high morbidity and mortality and increasing rates of hospitalization. The correlation between pulmonary microecology and infection severity remains unclear. Therefore, the purpose of this study was to investigate the differences in lung microecology and potential biomarkers in patients with mild and severe pulmonary infection.

**Method:**

Patients with pulmonary infection or suspected infection were divided into the mild group (140 cases) and the severe group (80 cases) according to pneomonia severity index (PSI) scores. Here, we used metagenomic next-generation sequencing (mNGS) to detect DNA mainly from bronchoalveolar lavage fluid (BALF) collected from patients to analyze changes in the lung microbiome of patients with different disease severity.

**Result:**

We used the mNGS to analyze the pulmonary microecological composition in patients with pulmonary infection. The results of alpha diversity and beta diversity analysis showed that the microbial composition between mild and severe groups was similar on the whole. The dominant bacteria were *Acinetobacter*, *Bacillus*, *Mycobacterium*, *Staphylococcus*, and *Prevotella*, among others. Linear discriminant analysis effect size (LEfSe) results showed that there were significant differences in virus composition between the mild and severe patients, especially Simplexvirus and Cytomegalovirus, which were prominent in the severe group. The random forest model screened 14 kinds of pulmonary infection-related pathogens including *Corynebacterium*, *Mycobacterium*, *Streptococcus*, *Klebsiella*, and *Acinetobacter*. In addition, it was found that *Rothia* was negatively correlated with *Acinetobacter*, *Mycobacterium*, *Bacillus*, *Enterococcus*, and *Klebsiella* in the mild group through co-occurrence network, while no significant correlation was found in the severe group.

**Conclusion:**

Here, we describe the composition and diversity of the pulmonary microbiome in patients with pulmonary infection. A significant increase in viral replication was found in the severe group, as well as a significant difference in microbial interactions between patients with mild and severe lung infections, particularly the association between the common pathogenic bacteria and *Rothia*. This suggests that both pathogen co-viral infection and microbial interactions may influence the course of disease. Of course, more research is needed to further explore the specific mechanisms by which microbial interactions influence disease severity.

## 1 Introduction

Pulmonary infection poses a significant threat to human health due to high morbidity and mortality rates (Diseases and Injuries, 2020). According to the 2019 statistical report of the World Health Organization (WHO), pulmonary infection is the fourth leading cause of death worldwide (Diseases and Injuries, 2020). In recent years, newly emerging respiratory pathogens such as influenza, middle east respiratory syndrome (MERS), and severe acute respiratory syndrome coronavirus 2 (SARS-CoV-2) have caused global pandemics, resulting in inflammation of the lung parenchyma, terminal airway, alveolar cavity, and lung interstitium. These pathogens have similar clinical symptoms, including fever, cough, sputum, chest pain, and even severe pneumonia with shock and organ failure (Zumla et al., 2015; Kevadiya et al., 2021; Cong et al., 2022). In the clinic, smear microscopy, culture, antigen/antibody testing, and nucleic acid are commonly used to detect the pathogens. However, more than 60% of lung infections remain unidentified and the severity of the condition cannot be determined (Miao et al., 2018). Recently, the rapidly developing metagenomic next-generation sequencing (mNGS) has been increasingly applied in the diagnosis and treatment of pulmonary infectious diseases, providing more microbial genotype information in the clinical setting (Miao et al., 2018; Li et al., 2020). mNGS maps the acquired sequence information to the microbial resource database, overcoming the limitations of targeted detection methods, and characterizes all microorganisms in the human system through a single detection. The rapid development of mNGS has increased our understanding of the diversity, composition, and function of the lung microbiome (Miao et al., 2022; Xiao et al., 2022; Vallianou et al., 2023).

The pulmonary microbiome is related to the maintenance of normal respiratory system function and the seriousness of lung disease (Fenn et al., 2022; Natalini et al., 2023). An imbalance in the lung microbiome community, leading to immune dysfunction in patients, may be a significant factor contributing to the occurrence and development of idiopathic pulmonary fibrosis and chronic obstructive pulmonary disease (COPD) (Invernizzi and Molyneaux, 2019; Ramsheh et al., 2021). Therefore, an in-depth study of lung microecology is conducive to further understanding of the pathogenesis of lung diseases and a search for new methods and means of prevention and treatment of lung diseases. Some articles summarized the composition and changes of pulmonary microbial communities, as well as the relationship between microorganisms and respiratory diseases in asthma (Huang and Boushey, 2015). The authors highlight the spatial variability of microbial communities in different parts of the lungs and establish a connection between different respiratory diseases and the composition of microbial communities (Dickson et al., 2013). Additionally, some studies reported that respiratory microbiome markers predict the occurrence or death of ventilator-associated pneumonia (VAP) in intensive care unit (ICU) patients (Mizrahi et al., 2017; Kitsios, 2018; Liu et al., 2022). However, there has been limited research investigating the link between respiratory microbiota and the severity of pneumonia, ranging from mild to severe cases.

To explore the composition of the pulmonary microbiome in patients with mild or severe infections, we used mNGS to evaluate the changes and characteristics of the microbiota in bronchoalveolar lavage fluid (BALF) or sputum. Additionally, we further analyzed the relationship between pulmonary microorganisms in different conditions with the analysis of alpha and beta diversity, linear discriminant analysis (LDA) effect size (LEfSe), random forest model, and microbial correlation.

## 2 Materials and methods

### 2.1 Recruitment of participants

**Figure 1:**
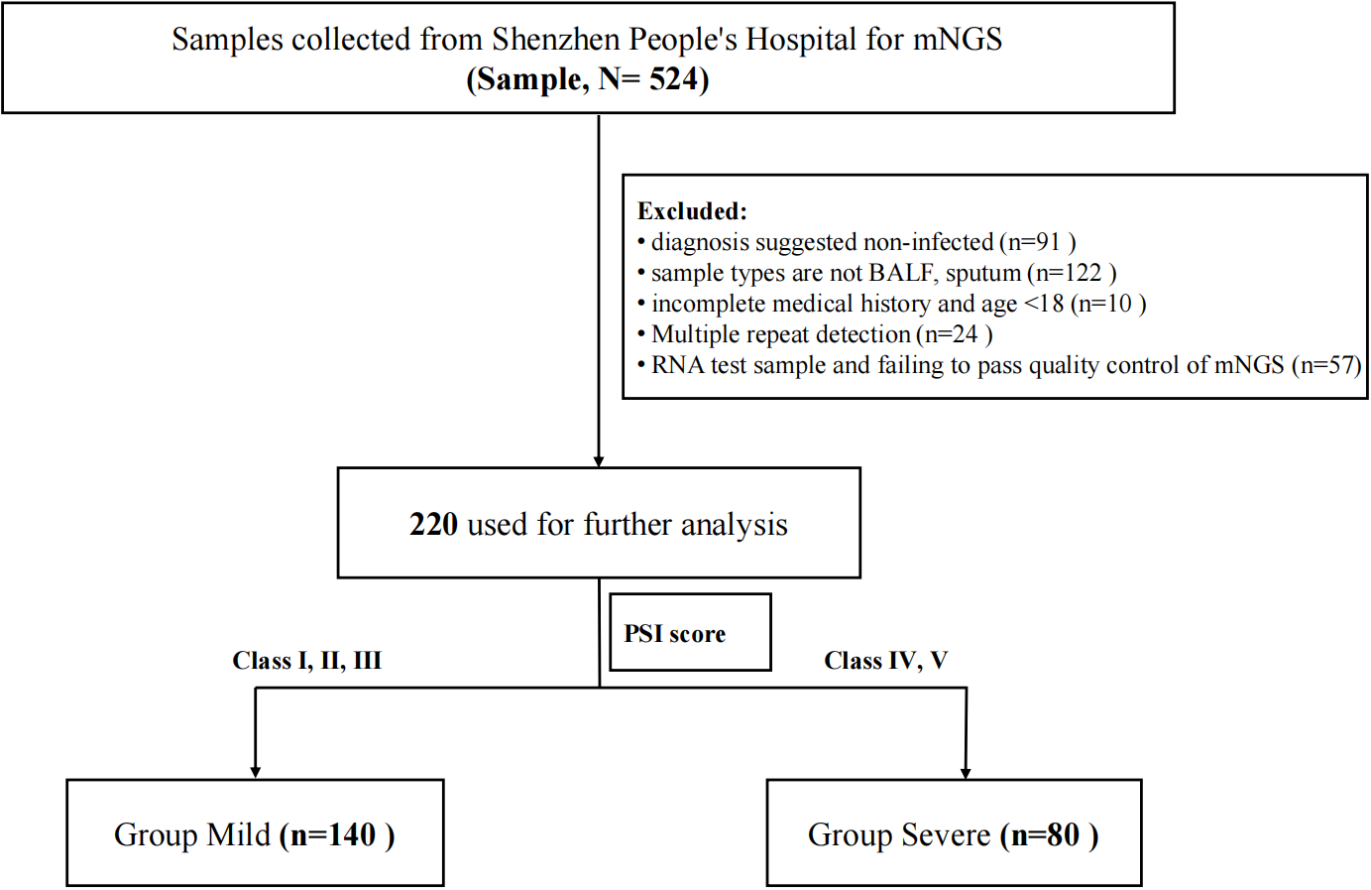
Flowchart.

This study is a retrospective study and was approved by Shenzhen People’s Hospital (LL-KY-2023129-01). Both samples and data were collected with the informed consent of the participants. Patients who were at least 18 years of age with pulmonary infection or suspected infection in Shenzhen People’s Hospital from 2019 to 2022 were enrolled. Pulmonary infection is defined according to the Chinese Guidelines for the Diagnosis and Treatment of community-acquired pneumonia in adults (Infectious Diseases Group R.S. and Chinese Medical Association 2018). Their BALF or sputum were collected and sent to BGI for mNGS. Clinical data were also collected from all enrolled patients for analysis. The exclusion criteria included (I) diagnosis suggested noninfected; (II) sample types are not BALF or sputum; (III) incomplete medical history and age <18; (IV) multiple repeat detection; (V) RNA test sample and failing to pass quality control of mNGS. The enrollment flowchart is shown in **Figure 1**.** **A total of 220 patients were finally included. They were divided into two groups based on PSI scores (Fine et al., 1997) that grade I, II, and III patients were defined as the mild group (n = 140) and grade IV and V patients were defined as the severe group (n = 80).

### 2.2 Definition

Smoking was defined according to the WHO, and these were patients who have smoked continuously or cumulatively for 6 months or more.

Respiratory failure: The patient feels that the air is insufficient and breathing is laborious. Objectively, there are strenuous breathing, mouth opening and shoulder shrug, nasal flap, cyanosis, auxiliary respiratory muscles are also involved in respiratory movement, and abnormal changes in respiratory frequency, rhythm, and depth.

Hypertension was diagnosed when baseline mean blood pressure was higher than clinically defined for both measurements (Systolic pressure ≥140 mmHg or Diastolic pressure ≥90 mmHg).

Diabetes was diagnosed when patients meet one of the following three criteria: 1) Fasting blood glucose reached or exceeded 7.0 mmol/L (126 mg/dL) twice on different days, and fasting was defined as no caloric intake for at least 8 h; 2) In the 75-g glucose tolerance test, blood sugar was higher than 11.1 mmol/L (200 mg/dL) at 2 h after a meal; 3) Have classic symptoms or of diabetes and random blood sugar above 11.1 mmol/L (200 mg/dL).

### 2.3 Sample collection and DNA extraction

According to the standard procedures, 1.5–3 mL of BALF or sputum from each enrolled patient was collected. In this study, 0.45-mL samples were mixed fully with saponin, which was at a final concentration of 0.025%, and incubated at 25°C for 5 min. Then, 75 μL was added for dehosting process, which was fully vortexed and incubated at 37°C for 10 min. After centrifugation at 18,000 g for 5 min, approximately 450 μL of supernatant was removed, and the final sample was retained at the bottom of ~70–80 μL. Then, 800 μL phosphate buffered saline (PBS) was added to the tube and fully vortexed followed by centrifugation at 18,000 g for 5 min. Then, approximately 800 μL supernatant was discarded, and the remaining ~70 μL–80 μL at the bottom was mixed with 370 μL TE-buffer followed by shaking. For the wall-breaking reaction, 7.2 μL lysozyme was added. Here, 250 μL of 0.5-mm glass beads were attached to a horizontal platform on a vortex mixer and agitated vigorously at 2,800 rpm–3,200 rpm for 30 min. Then, 0.3-mL sample was separated into a new 1.5-mL microcentrifuge tube, and DNA was extracted using the TIANamp Micro DNA Kit (DP316, TIANGEN BIOTECH) according to the manufacturer’s recommendation.

### 2.4 Construction of DNA libraries and sequencing

DNA libraries were constructed through DNA fragmentation, end-repair, adapter ligation, and PCR amplification. Agilent 2100 was used for quality control of the DNA libraries. Quality-qualified libraries were pooled, and the DNA Nanoball (DNB) was made and sequenced by MGISEQ-2000 platform (Jeon et al., 2014).

### 2.5 Bioinformatic analysis

We first used fastp to filter the raw sequencing data (Chen et al., 2018). Then, we used hisat2 (version 2.2.1) for mapping the reads to the human genome GRCh38 with default parameters and removed the mapped reads (Kim et al., 2019). Finally, microbial species were identified based on the analysis of the clean reads by Kraken2 (Wood et al., 2019). Values for the alpha diversity (Shannon’s index, Simpson index, and Richness), beta diversity, and principal coordinate analysis (PCoA) and nonmetric multidimensional scaling (NMDS) based on the Bray–Curtis metrics were generated by R (version 4.2.2). Venn diagram showing the number of common and unique operational taxonomic units (OTUs) between the two groups was made by an online tool (http://bioinformatics.psb.ugent.be/webtools/Venn/). LEfSe was used to determine the features that most likely explain the differences between the groups (http://huttenhower.sph.harvard.edu/galaxy/root?tool_id=PICRUSt_normalize). The random forest model by R was used to screen the key bacteria that distinguished the two groups of samples. The network of microorganisms was produced by retaining edges (correlation coefficient R ranges between −0.6 and 0.6 and p < 0.05), analyzed in R with the package igraph and visualized with Gephi 0.10.0.

### 2.6 Statistical analysis

In order to test the significant correlation between clinical features and disease, continuous variables were analyzed with Mann–Whitney test and categorical variables were analyzed with chi-square test. All above analyses were done by GraphPad Prism 7.0. Wilcoxon signed rank test was used to compare alpha diversity measures. PERMANOVA was used to compare beta diversity measures.

## 3 Results

### 3.1 Clinical characteristics

More than 40M sequencing reads were generated for mNGS analyses for which microorganisms accounted for approximately 1%. After quality control filtering, removal of potential human DNA contamination and the samples that had <5,000 sequences, a total of 220 recipients with pulmonary infection or suspected infection were enrolled in this study finally, including 80 of the 220 patients into the severe group and 140 into the mild group according to the PSI scores (**Figure 1**). Patient characteristics are summarized in **Table 1**. There were more men [76 men (54.29%) in mild cases and 66 men (82.5%) in severe cases] than women in this cohort. The age of mild patients is generally younger than that of severe patients. The median age of the mild group was 48.5 (IQR 39–57) years old, and for the severe group, it was 71 (IQR 65–78) years old. For clinical symptoms, the number of patients with respiratory failure were significantly higher in the severe group than that in the mild group. We have also noticed a higher number of severe patients with underlying diseases such as tumor, COPD, coronary heart disease, and diabetes. Blood routine can reflect human immunity to a certain extent, but the level of immunity needs to be combined with the actual situation of the patient and other examinations to make a comprehensive judgment.

**Table 1:** Clinical characteristics.

Characteristics (Median [IQR] or n [%])	Mild (n = 140)	Severe (n = 80)	p value
Age	48.5 [39-57]	71 [65-78]	<0.0001
Male	76 [54.29]	66 [82.5]	<0.0001
Clinical symptoms			
Cough	94 [67.14]	51 [63.75]	0.6096
Sputum	74 [52.86]	45 [56.25]	0.6271
Hemoptysis	9 [6.43]	7 [8.75]	0.5236
Respiratory failure	8 [5.71]	21 [26.25]	<0.0001
Others	4 [2.85]	9 [11.25]	0.0111
Physical examination findings			
Body temperature (**°C**)	36.7 [36.4-38]	37.4 [36.5-38.73]	0.0152
Pulse (times/min)	90.5 [81-102]	92 [80.75-102.5]	0.6531
Respiratory rate (times/min)	20 [20-22]	22 [20-22]	0.0517
Systolic blood pressure (mmHg)	120 [108.75-131.25]	127 [116.75-139.5]	0.0071
Underlying disease			
Smoking	32 [22.86]	39 [48.75]	<0.0001
Tumor	5 [3.57]	15 [18.75]	0.0002
Diabetes	7 [5]	18 [22.5]	<0.0001
COPD	2 [1.42]	9 [11.25]	0.0013
Hypertension	22 [15.71]	19 [23.75]	0.1409
Coronary heart disease	0 [0]	6 [7.5]	0.0010
Others	1 [0.71]	7 [8.75]	0.0022
Others			
General ward days	10 [7-16.25]	13 [9.75-19.5]	0.025
Total hospital days	11 [8-17.25]	16.5 [11-27.25]	<0.0001
Cost	18268.78 [12766.62-35050.83]	44417.6 [19725.72-87830.86]	<0.0001

**Figure 2:**
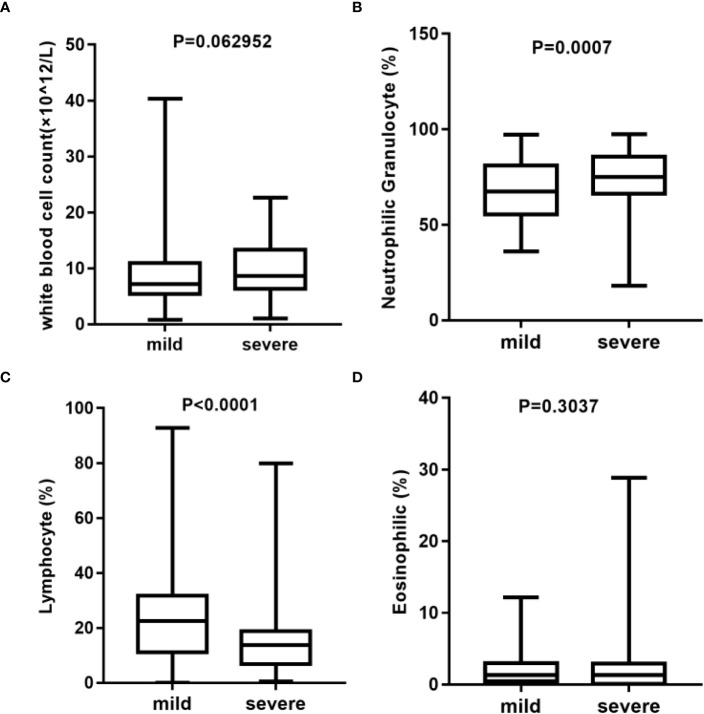
Analysis of blood routine between mild (n = 137) and severe (n = 78) group*. **(A)** Statistical analysis of white blood cell count. **(B)** Statistical analysis of neutrophilic granulocyte percentage. **(C)** Statistical analysis of lymphocyte percentage. **(D)** Statistical analysis of eosinophilic percentage. *Blood routine data were missing in three cases in the mild group and in two cases in the severe group.

Here, we analyzed the main indicators of immunity in blood routine with total white blood cells (**Figure 2A**), neutrophilic granulocyte percentage (**Figure 2B**), lymphocyte percentage (**Figure 2C**), and eosinophilic percentage (**Figure 2D**), among others. The results showed that there were significant differences in the percentage of neutrophilic granulocyte and lymphocytes between the mild and severe groups. There was no statistically significant difference of C-reactive protein (CRP) (p = 0.1447) and Interleukin (IL-6) (p = 0.5267) in the two groups (**Supplementary Figures S1A, B**). However, the procalcitonin (PCT) (p = 0.0237) in the severe group was significantly higher than that in the mild group (**Supplementary Figure S1C**). To be noted, as the study is a retrospective research, not all included patients underwent CRP, PCT, and IL-6 testing. These results suggest that there may be differences in the immune status between the two groups.

### 3.2 Microbial composition in patients with mild or severe pulmonary infection

**Figure 3:**
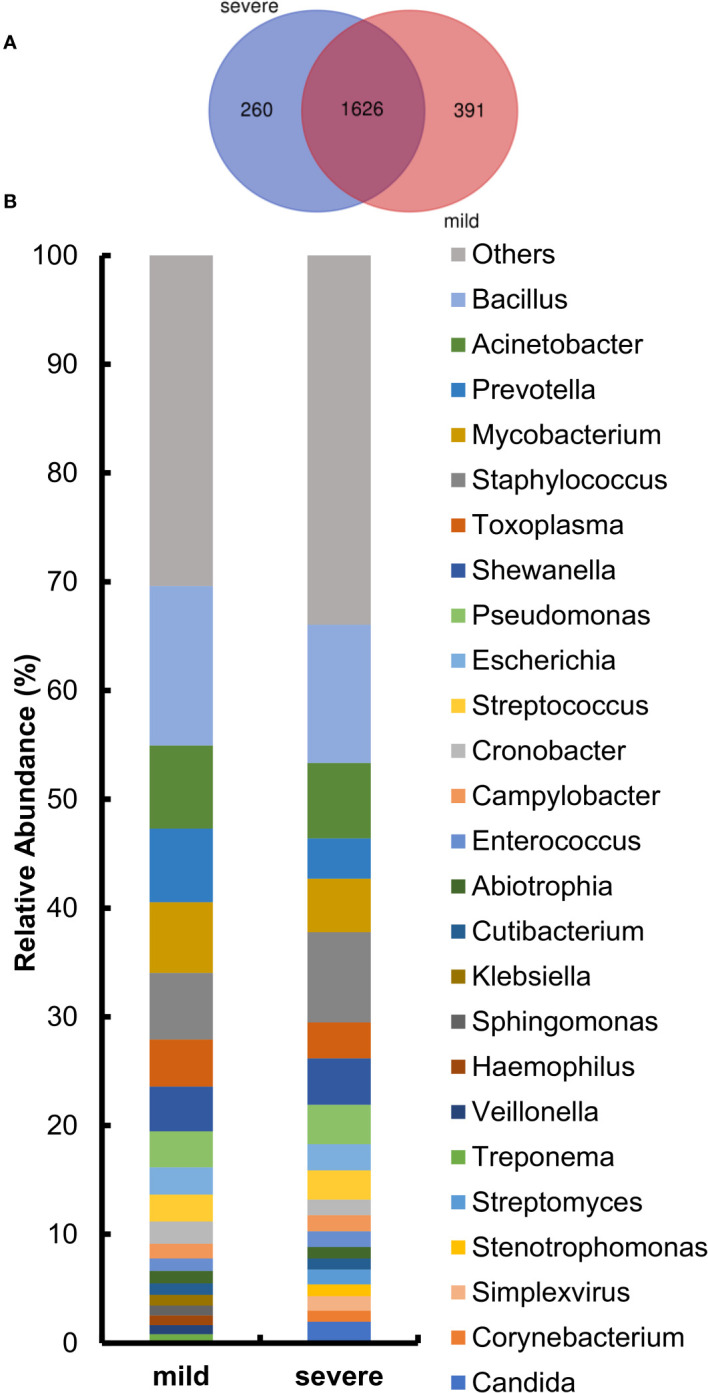
Comparison of relative abundance at the genus level between the mild and severe groups. **(A)** The Venn diagram based on the total microorganisms of the patients. **(B)** Pulmonary microbiome composition at the genus level.

After the identification of microorganisms and the bacterial genera below 10 reads were removed, the diversity and species composition were analyzed. The abundance level is based on the relative percentage of reads, and the analysis of alpha/beta diversity is based on the reads table. In this study, 15 of the 220 samples had a history of tumor. To exclude the effect of the tumor on the overall analysis, the microbial diversity of tumor patients and non-tumor patients was analyzed. The results showed that there was no statistically significant difference between tumor patients and non-tumor patients in alpha and beta diversity (**Supplementary Figure S2**). For another, the proportion of patients with a history of tumor is relatively small and these patients had not been treated with chemotherapy for nearly a month, so we did not specifically distinguish this category. For the 220 samples, we first compared the composition of lung microorganisms among the mild and severe groups. As shown in **Supplementary Figures S3A, B**, the alpha diversity with Shannon index and Simpson index revealed no significant differences between the mild and severe subjects. Similarly, the analysis of the beta diversity calculated with PCoA and NMDS based on the Bray–Curtis metrics also showed no difference in the two groups (**Supplementary Figures S3C, D**). These results suggest that there is no significant difference in overall microbial diversity between mild and severe patients. Moreover, a Venn diagram of bacteria showed that 1,626 of the total 1,977 genera were shared among the two groups, while 260 genera were unique for the severe group and 391 genera were unique for the mild group (**Figure 3A**). For the two groups at the genus level, the relative abundance of the top 20 genera was analyzed (**Figure 3B**). *Acinetobacter*, *Bacillus*, *Mycobacterium*, *Staphylococcus*, and *Prevotella* accounted for a high proportion of patients in both groups.

### 3.3 Bacterial biomarkers in the mild and severe patients

**Figure 4:**
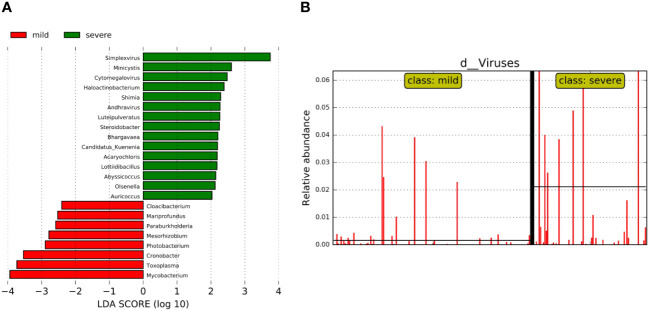
Bacterial biomarkers were identified by linear discriminant analysis effect size (LEfSe) algorithm. **(A)** Bacterial histograms of unique biomarkers based on LEfSe (>2). The length of the bar chart represents the magnitude of the impact of significantly different genus. **(B)** Differences in the relative abundance of viruses between the two groups.

We further analyzed the bacterial community structure associated with the mild and severe groups using LEfSe, an algorithm for high abundance biomarker discovery that uses LDA to estimate the effect size of each taxon that differed between the two groups (**Figure 4**). A total of 23 distinct genera were identified. For the mild group, there were eight identified potential markers, mainly including *Mycobacterium*, *Toxoplasma*, and *Cronobacter* (**Figure 4A**). Similarly, we observed a significant increase in the relative abundance of the virus in the severe group compared to the mild group (**Figure 4B**). The potential markers for the severe group included Simplexvirus and Cytomegalovirus (CMV) (**Figure 4A**). Mycobacterium and Simplexvirus had the highest LDA scores, indicating a strong influence of microbial relative abundance in the mild group and severe group, respectively.

### 3.4 High-contribution bacteria selected by random forest model

**Figure 5:**
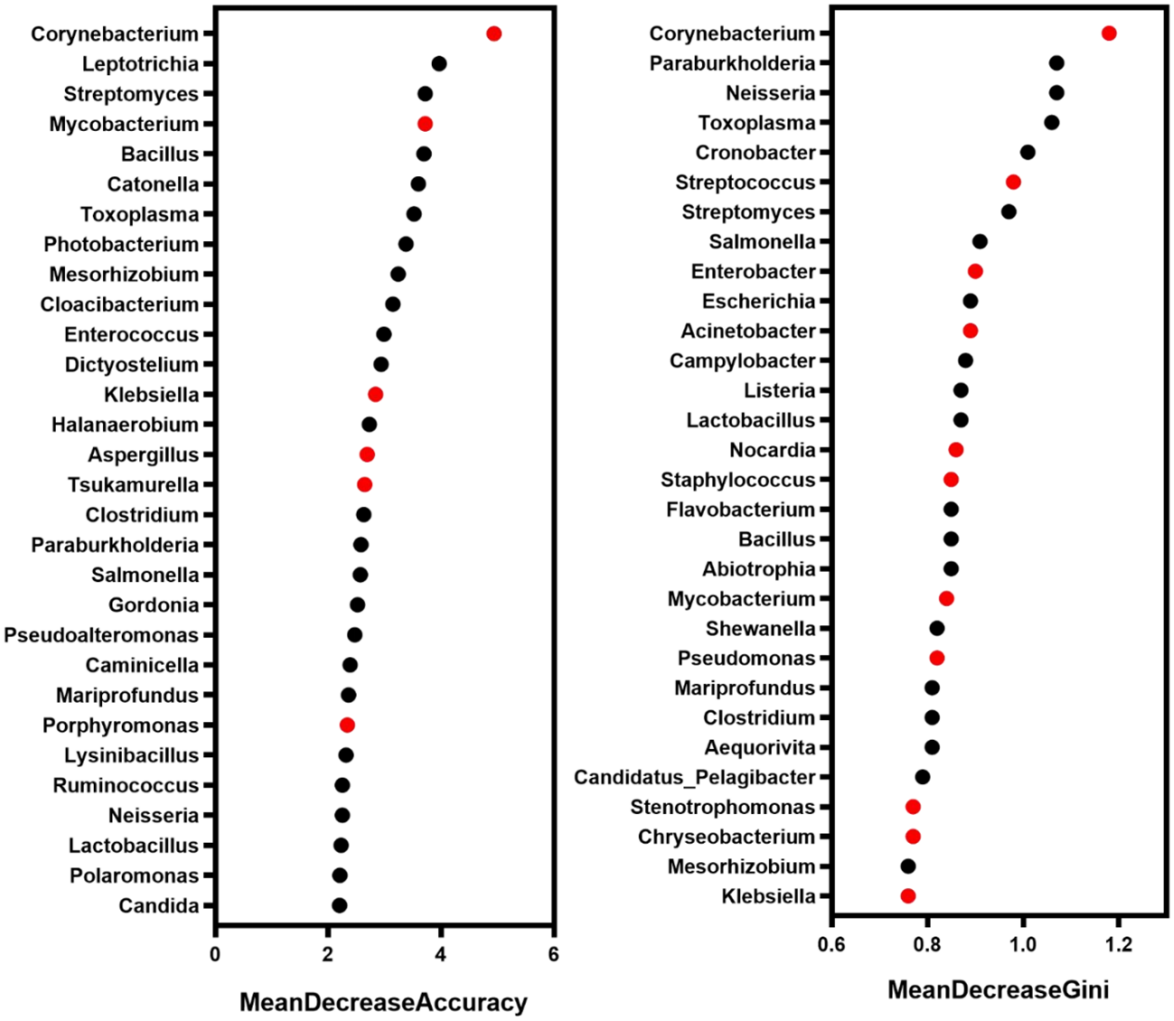
The microbe with a high contribution to distinguishing the severity of infection. Red marker: the microbe associated with pulmonary infection.

In order to more comprehensively describe the microbial characteristics between the mild group and the severe group, 30 key pathogenic bacteria were screened by random forest model with the analysis of MeandecreaseAccuracy and MeandecreaseGini, respectively. Combined with the pathogenic characteristics of these microorganisms, we finally found 14 bacteria that included *Acinetobacter*, *Mycobacterium*, and *Klebsiella* with a greater contribution to distinguishing patients with mild and severe pulmonary infection (**Figure 5**).

### 3.5 Significant differences in microbial interaction modes in the mild and severe groups

**Figure 6:**
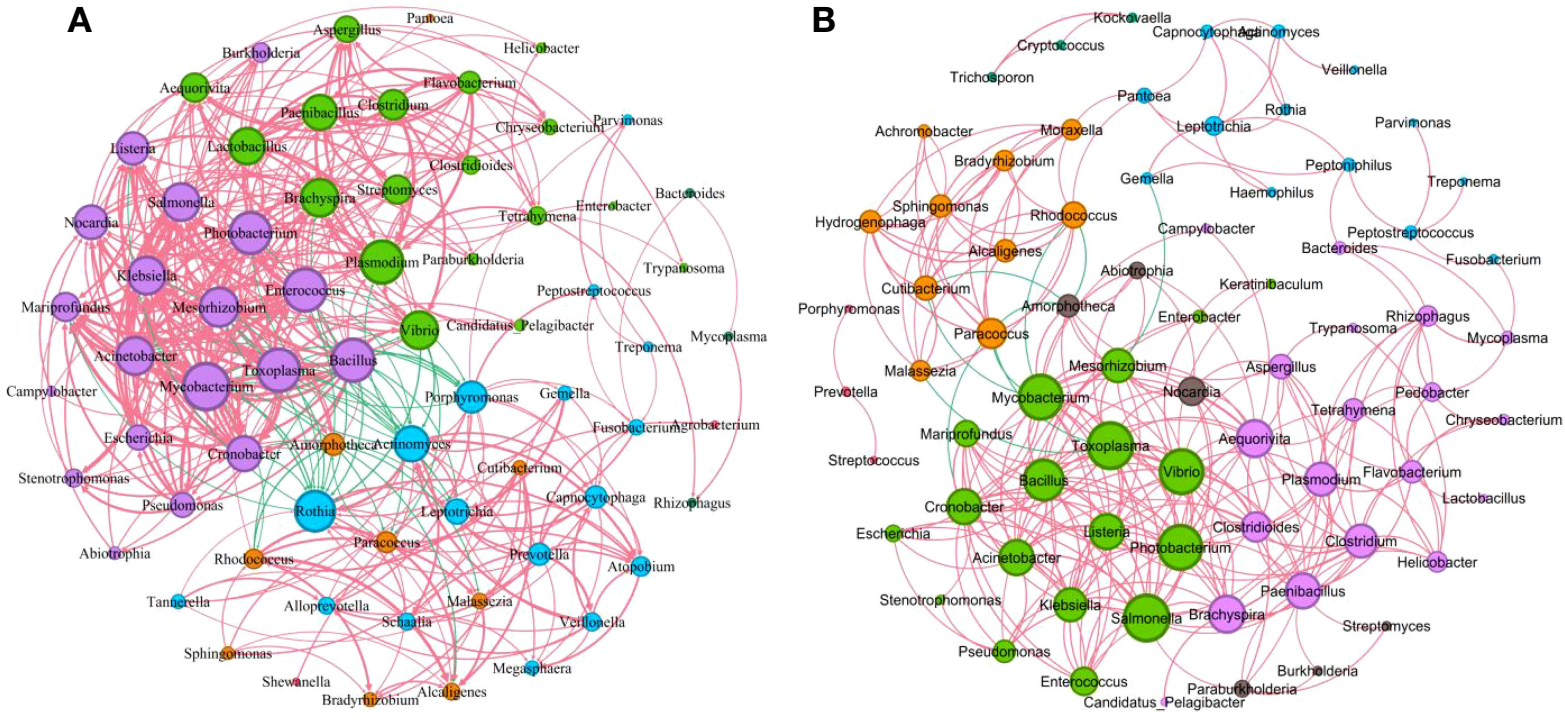
Correlations between key microorganisms in the two groups. **(A)** Network co-occurrence diagram between microbes (mild). **(B)** Network co-occurrence diagram between microbes (severe). Circles of the same color belong to one module, and the size of each circle represented the degree (refers to the number of nodes directly connected to a node, reflects the ecological role of a particular microbe). The red line indicates a positive correlation between microbes, while the green line indicates a negative correlation. The thickness of the line reflects the size of the Spearman coefficient between two species. The thicker the line, the higher the correlation.

We further performed the network co-occurrence map with Spearman correlation analysis based on the bacteria with relative abundance greater than 0.1% in the mild group (83 genera) and the severe group (89 genera), respectively (**Supplementary Figure S4**). As shown in **Figure 6**, the microbial network of the mild group consisted of 68 nodes and 431 edges (389 with a positive correlation and 42 with a negative correlation), which is more complicated than the network with 72 nodes and 294 edges (284 with a positive correlation and 10 with a negative correlation) of the severe group. In the mild group (**Figure 6A**), the central genus in this network is *Mycobacterium*. There is a strong positive correlation between *Toxoplasma* and *Cronobacter*, *Mycobacterium* and *Mesorhizobium* among others, while in the severe group (**Figure 6B**), *Toxoplasma* and *Salmonella* were the central genera. There is a strong positive correlation between *Alcaligenes* and *Hydrogenophaga*, *Mycobacterium* and *Toxoplasma* among others. Interestingly, we found that *Rothia* was negatively associated with *Acinetobacter*, *Mycobacterium*, *Bacillus*, *Enterococcus*, *Klebsiella*, and other pulmonary infection-related pathogens in the mild group but not significantly associated with those pathogenic bacteria in severe cases (**Figures 6A, B**). These results suggest that differences in microbial interactions between patients with mild and severe pulmonary infections may be responsible for the differences in disease severity.

## 4 Discussion

The pulmonary microbiome could reflect the maintenance of normal respiratory system function and the seriousness of lung disease. However, there is little research on the microbiome with the severity of pneumonia. Here, we analyzed the specific microbial composition of the mild and severe pneumonia groups with the analysis of alpha and beta diversity and screened the biomarkers of patients with lung infections with varying severity by LEfSe and random forest model. Finally, the correlation between microorganisms was analyzed with the selected biomarkers. In diversity analysis in the two groups, our results showed that the top 20 genera with relative abundance in the mild group accounted for 69.6% of the total abundance. Specifically, the top 5 genera were *Bacillus*, *Acinetobacter*, *Prevotella*, *Mycobacterium*, and *Staphylococcus*. In contrast, the top 20 genera in the severe group accounted for 66.06% of the total, and the top 5 were *Bacillus*, *Staphylococcus*, *Acinetobacter*, *Mycobacterium*, and *Shewanella*. Which is consistent with the previously reported, the taxonomic groups account for pulmonary infection in patients with lung microecological dominance (Chen et al., 2021; Fenn et al., 2022). Although *Bacillus* has rarely been reported as a direct cause of lung infection, it has been shown to contribute to the development of severe pneumonia and is considered a potential pathogen (Shimoyama et al., 2017). LEfSe analysis and random forest model could be used for feature selection and biomarker screening. For example, LEfSe analysis identified the major genera in the pneumonia group as *Pseudomonas*, *Corynebacterium*, *Roche*, *Enterococcus*, and *Neisseria* (Woo et al., 2020). Random forest model selected pneumonia *Klebsiella* bacteria and *Bacillus* wax samples as community-acquired pneumonia patients with high AUC value potential diagnostic biomarkers (Hong et al., 2021). Here, we used LEfSe and random forest model respectively to further analyze the differentially expressed microorganisms between the two groups. LEfSe analysis identified 23 different genera in mild and severe patients, of which eight genera including *Mycobacterium*, *Toxoplasma*, and *Cronobacter* were dominant in the mild group, while 15 genera including Simplexvirus, *Minicystis*, and CMV were dominant in the severe group. In addition, the relative abundance of virus in the severe group was significantly higher than that in the mild group. CMV reactivation causes serious consequences in non-immunosuppressed critical surgery patients, and pneumonia caused by CMV infection has a higher morbidity and mortality rate in immunocompromised individuals (Huang and Tang, 2021). It has previously been reported that quantification of CMV viral load in BALF can be used to assist in the diagnosis of CMV pneumonia (Styczynski, 2018). Similarly in patients with VAP, highly replicative Herpes simplex virus (HSV) has been shown to play a pathogenic role in patients (Schuierer et al., 2020). We guessed whether the severity of pneumonia was related to the relative abundance of virus, but more evidence is still needed. Based on the random forest model, we finally screened out 14 bacterial genera that may be related to the occurrence of pulmonary infection, including *Corynebacterium*, *Mycobacterium*, *Streptococcus*, *Klebsiella*, and *Acinetobacter*. These pathogens are common pathogens associated with lung infections (Sharma et al., 2019; Fenn et al., 2022; Griffith and Daley, 2022).

In addition to single microbial infections, the interactions between microorganisms are receiving increasing attention from researchers (Huang et al., 2022; Zhang et al., 2022). In this study, we screened bacterial genera with relative abundance greater than 0.1%, conducted correlation analysis for each genus, and visualized the results through a network diagram. Our findings indicated that the network map was more complex in the mild group than that in the severe group. Moreover, we observed negative relationships between *Rothia* and several pulmonary infection-related pathogens, including *Acinetobacter*, *Mycobacterium*, *Bacillus*, *Enterococcus*, and *Klebsiella*, in the mild group. However, in the severe group, *Rothia* was not significantly correlated with these pathogens. Previous studies have shown that *Rothia* plays a role in the pathogenesis of respiratory tract infections (Laufer et al., 2011; Ramanan et al., 2014). Based on our findings, we speculate that the interaction between *Rothia* and these lung infection-associated pathogens may contribute to the severity of the infection. Further studies are needed to validate this hypothesis and explore the underlying mechanisms.

However, there are still some limitations in this study. First, it only analyzed patients from a single center, and the lung microbiome may be related to underlying diseases, habits, and geographic origin; so, further screening of biomarkers in a wider population should be done. Second, this study only explored the microbial differences and associations among patients with mild and severe pulmonary infection based on the results of mNGS analysis, without conducting experimental studies to further confirm the influencing mechanism.

In summary, our study investigated the composition and diversity of the pulmonary microbiome in patients with mild and severe lung infections. We identified biomarkers that distinguish between mild and severe cases and further analyzed the association between microbial interactions and disease severity. Our findings suggest that *Rothia* is negatively associated with common lung infection-associated pathogens in mild cases and positively or not significantly associated in severe cases. However, further research is necessary to elucidate the specific mechanism of microbial interaction on disease severity in the future.

## Data availability statement

The data that support the findings of this study have been deposited into CNGB Sequence Archive (CNSA) of China National GeneBank DataBase (CNGBdb) with accession number CNP0004655 upon reasonable request. You can follow the link (https://db.cngb.org/search/project/CNP0004655/) to see the details.

## Ethics statement

The studies involving humans were approved by Research Ethics Committee of Shenzhen People’s Hospital. The studies were conducted in accordance with the local legislation and institutional requirements. The human samples used in this study were acquired from a by- product of routine care or industry. Written informed consent for participation was not required from the participants or the participants’ legal guardians/next of kin in accordance with the national legislation and institutional requirements.

## Author contributions

LW, JZ, DZ, KY and DL contributed to conception and design of the study. LW, JZ and DZ collected clinical data and organized the database. YS, LH and DL performed the figure and statistical analysis. DL wrote the first draft of the manuscript. LW, DZ, KY wrote sections of the manuscript. All authors contributed to manuscript revision, read, and approved the submitted version.
