# Supplementary material for: Characteristics of the pulmonary microbiota in patients with mild and severe pulmonary infection
Source: Front Cell Infect Microbiol. 2023 Oct 12;13:1227581. doi: 10.3389/fcimb.2023.1227581 (PMC10602873; doi:10.3389/fcimb.2023.1227581)
Supplement: Supplementary file 1 [file DataSheet_1.pdf]

Supplementary Material

Characteristics of the pulmonary microbiota in patients with mild and severe pulmonary infection

Danting Zhan1†, Dan Li2†, Ke Yuan2†, Yihua Sun2, Lijuan He2, Jiacheng Zhong1*, Lingwei Wang1*

[1]Shenzhen Institute of Respiratory Diseases, Shenzhen People's Hospital, Guangdong, China

[2]BGI Genomics, ~~BGI-Shenzhen,~~ Shenzhen 518083, China

*** Correspondence:**

Corresponding Author: Jiacheng Zhong, zhong.jiacheng@szhospital.com; Lingwei Wang, limey@sina.com

†These authors contributed equally to this work and share first authorship

Supplementary Figures

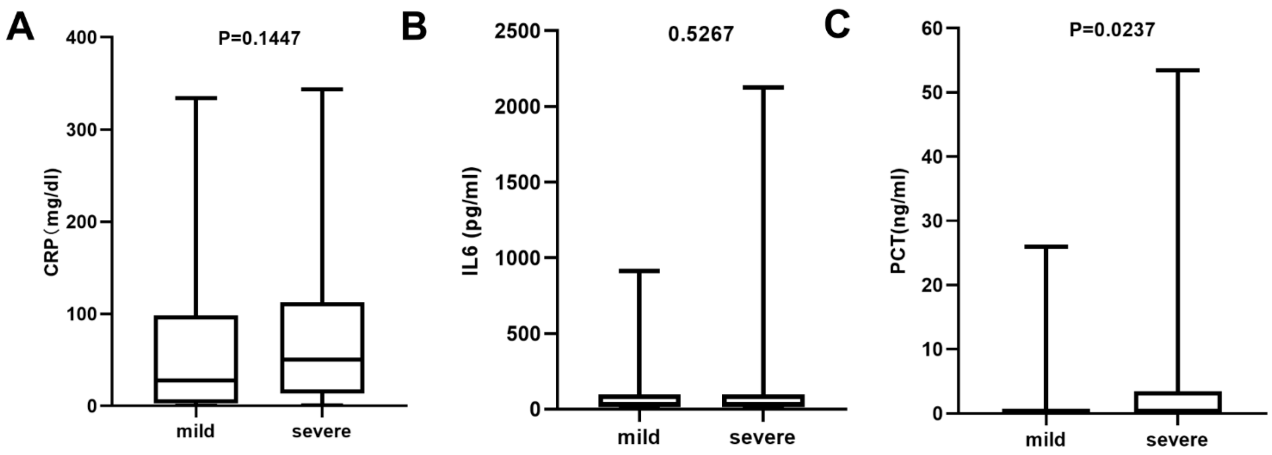

Supplementary Figure 1. Analysis of blood routine between mild and severe group. (A) Statistical analysis of C-reactive protein [CRP, Mild (N=91) vs. severe (N=63)]. (B) Statistical analysis of IL-6 [Mild (N=38) vs. severe (N=27)]. (C) Statistical analysis of Procalcitonin [PCT, Mild(N=55), severe(N=45)].

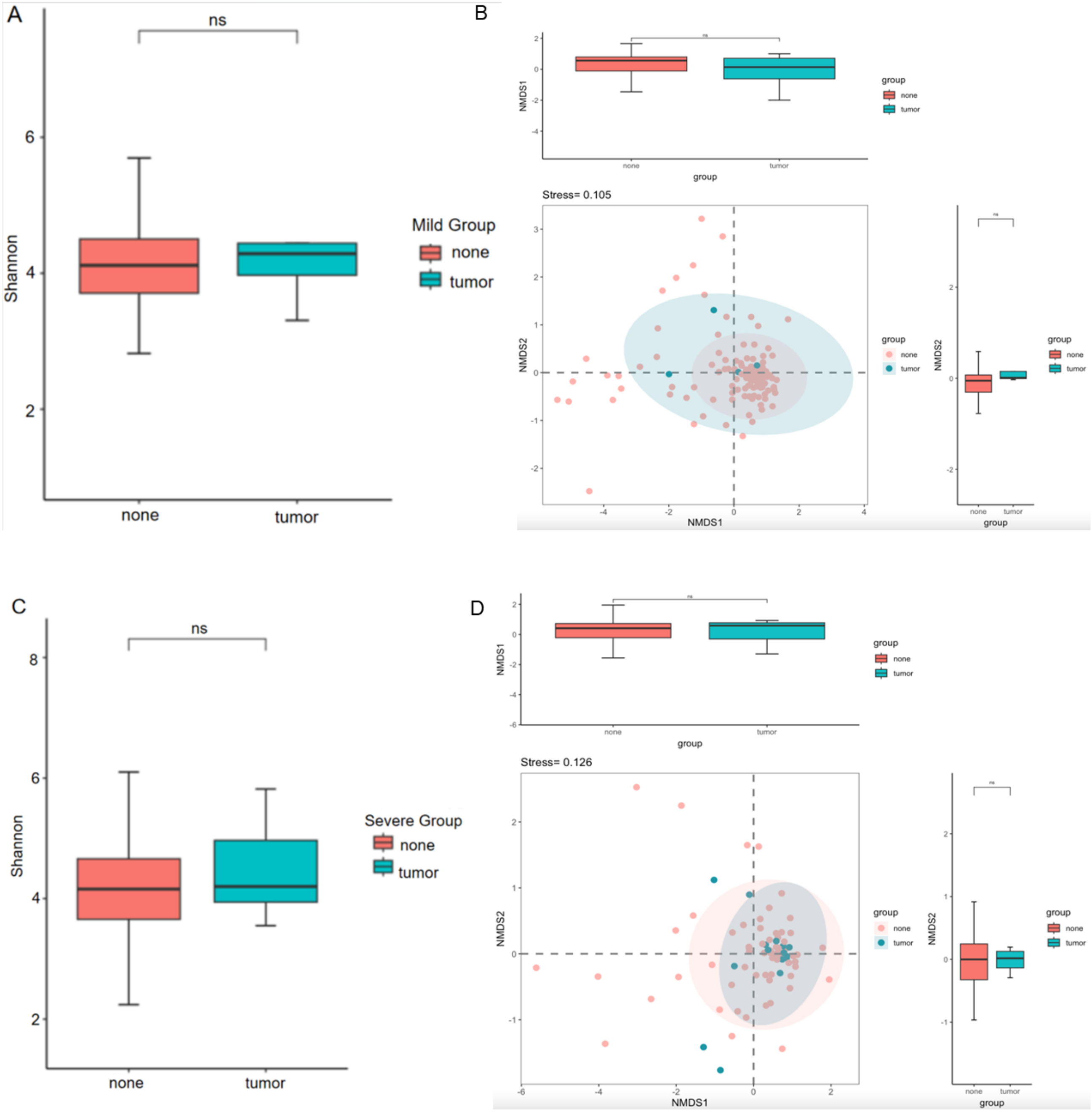

Supplementary Figure 2. Diversity of bacterial in tumor and non-tumor patients. (A-B) alpha diversity of Shannon diversity index and beta diversity between tumor and non-tumor patients at mild group. (C-D) alpha diversity of Shannon diversity index and beta diversity between tumor and non-tumor patients at severe group

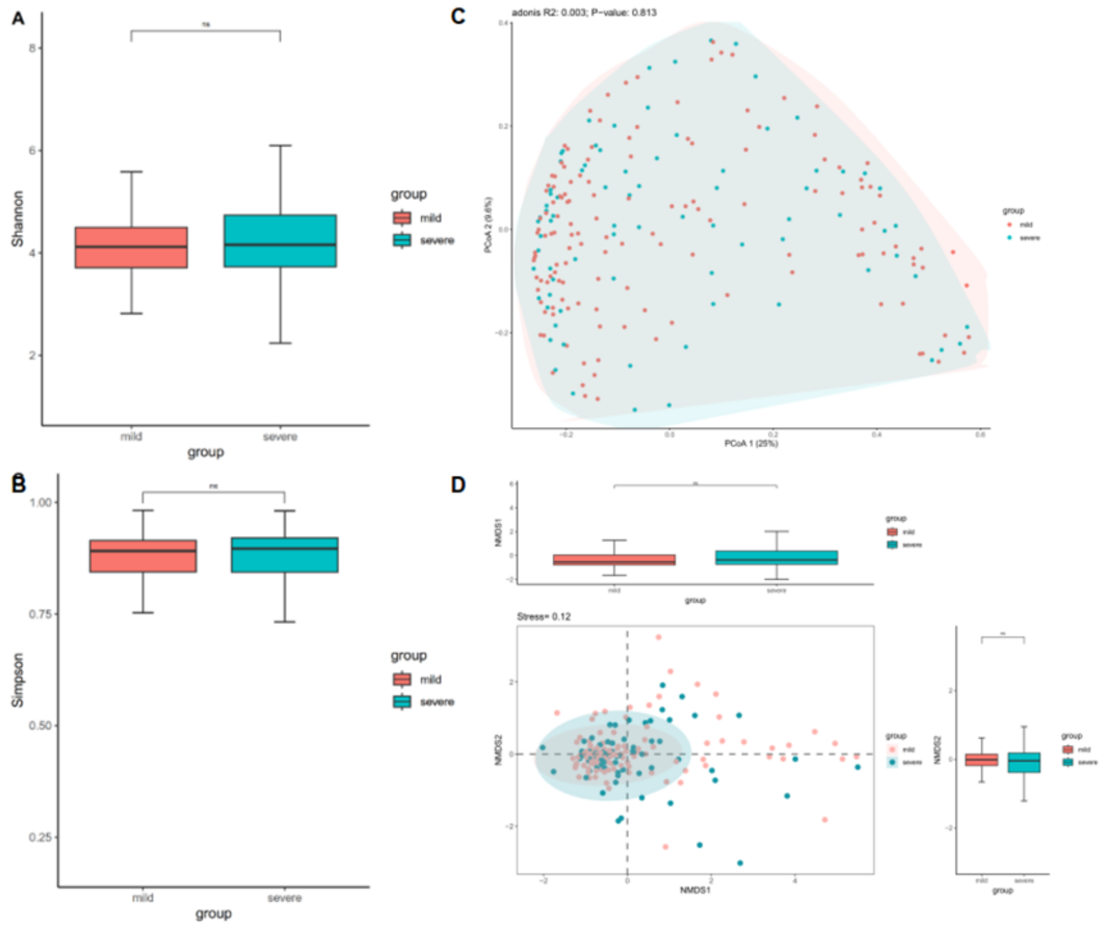

Supplementary Figure 3~~1~~. Diversity of bacterial in patients with pulmonary infection. (A-B) alpha diversity of Shannon diversity index and Simpson index. (C) Bray-Curtis distance of the pulmonary microbiota, shown as a principal coordinate analysis (PCoA) two-dimensional map. Each dot represents one sample. (D) Bray-Curtis distance of the pulmonary microbiota, shown as a Non-metric multidimensional scaling (NMDS). Each dot represents one sample.

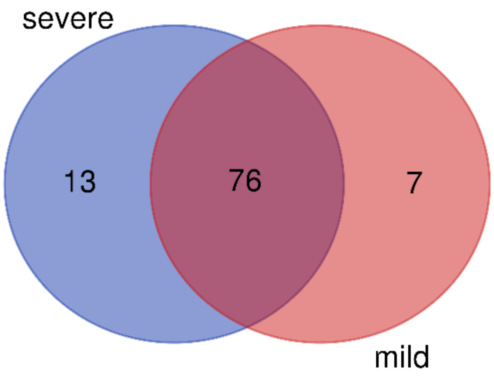

Supplementary figure 4. The Venn diagram based on the bacteria with a total abundance of more than 0.1%.
